# Exploring Inhibition Mechanism of Si on Cementite Nucleation in Hypereutectoid Steel: Experiments and First-Principles Calculations

**DOI:** 10.3390/ma17010223

**Published:** 2023-12-30

**Authors:** Taixu Xu, Zhijun He, Xiao Han, Xin Yang, Xinmei Hou

**Affiliations:** 1 School of Materials and Metallurgy, University of Science and Technology Liaoning, Anshan 114051, China; 2 Key Laboratory of Green Low-Carbon and Intelligent Metallurgy Liaoning Province, University of Science and Technology Liaoning, Anshan 114051, China; 3 Collaborative Innovation Center of Steel Technology, University of Science and Technology Beijing, Beijing 100083, China

**Keywords:** cementite nucleation, Si concentration, γ-Fe/Fe_3_C interfaces, interface stability, electronic structure

## Abstract

To clarify the influence of Si on cementite nucleation during the solidification of hypereutectoid steel, the types and microstructure of cementite in hypereutectoid steel with various Si concentrations were investigated by X-ray diffraction and scanning electron microscopy. Additionally, the interfacial properties of γ-Fe/Fe_3_C were studied using the first-principles density functional theory, including work on adhesion, interfacial energy, and electronic structure, with the aim of elucidating the impact mechanism of Si on the cementite nucleation. The results showed that increasing Si concentrations (0–0.42 wt.%) had a negligible effect on the types of cementite in as-cast hypereutectoid steel. However, the average number of cementite lamellae per unit area decreased significantly, indicating that an increase in Si concentrations has an inhibitory effect on cementite nucleation. This can be attributed to the effect of Si on the interfacial properties of γ-Fe (010)/Fe_3_C (010), where the presence of Si disrupts the charge distribution of the γ-Fe (010)/Fe_3_C (010) interface and decreases the hybridization of atom orbits on each side of the interface, resulting in a decrease in the interatomic interaction force. This is reflected in the decrease in the work of adhesion (from 6.92 J·m^−2^ to 6.78 J·m^−2^) and the increase in the interfacial energy (from −1.42 J·m^−2^ to −1.31 J·m^−2^). As a result, the stability of the γ-Fe (010)/Fe_3_C (010) interface is reduced, making it difficult for the composite structure to form. This indicates that Si doping inhibits cementite nucleation on austenite.

## 1. Introduction

Cementite plays a vital role in the mechanical properties of hypereutectoid steel due to its high hardness and brittleness [1,2,3]. Therefore, controlling the nucleation and growth of cementite is particularly important for optimizing the properties of hypereutectoid steel. The control of cementite nucleation cannot only affect the amount of cementite, but can also indirectly change the growth of cementite [4,5], representing a research topic that has attracted extensive attention.

The factors affecting cementite nucleation are the cooling rate, deformation, and addition of alloy elements, which have been widely studied [6,7,8]. Nevertheless, the influence of the non-metallic elements in steel on cementite nucleation has not been sufficiently considered, especially the non-carbide-forming element Si, which has only been discussed in a few studies. Kim et al. pointed out that the increase in Si concentration decreased the driving force of cementite nucleation in a nonequilibrium state [9]. Kozeschnik et al. found that when Si remained in cementite and the nucleation tendency was low, Si might postpone cementite nucleation [10]. The aforementioned research results indicated that Si can inhibit cementite nucleation, and this inhibition effect can give more variable operations during the heat treatment process [11,12]. However, these studies were not sufficiently thorough and mostly focused on the microstructure level to analyze the influence of Si on cementite nucleation. Consequently, it is necessary to interpret the nucleation potential of Si affecting cementite at the atomic scale. Although Jang et al. reported the influence of Si on the precipitation of cementite from a microscopic perspective using a density functional theory, their research assumed that Si facilitated the nucleation of *ε*-carbide via a coherent effect [13]. The results appear to be rather counterintuitive. Hence, the influence of Si on cementite nucleation must be clarified, thereby allowing for greater flexibility in designing the Si concentration in steel.

First-principles calculations based on the density functional theory have been extensively applied to simulate and analyze the interfacial properties between different phases [14,15,16]. Additionally, for the mechanisms that are difficult to access experimentally because of the limitations of the testing methods, the first-principles calculation can give a more intuitive explanation through the change in atomic energy and the change in electron migration; for instance, revealing the mechanism of TiC as an effective ferrite [17], exploring the priority of NbC heterogeneous nucleation on TiN [18], and elucidating the impact mechanism of rare-earth elements on the D019-Co3X detrimental phase [19]. Consequently, the mechanism of Si on cementite nucleation on austenite can be further explored from an atomic perspective through first principles.

In this work, the phases of hypereutectoid steels with various Si concentrations (0–0.42 wt.%) were determined through thermodynamic calculation and X-ray diffraction (XRD). The microstructure of the cementite in the as-cast hypereutectoid steel with various Si concentrations was observed through scanning electron microscopy (SEM). In addition, in order to reveal the essence of Si affecting cementite nucleation, the first principles were used to calculate the effect of Si on the austenite–cementite interface. First, the orientation relationship between cementite and austenite in the experimental steel were determined through electron backscatter diffraction (EBSD), and the computational models of austenite–cementite (γ-Fe/Fe_3_C) were finally determined by calculating the surface energy. Then, based on first-principles calculations, the interfacial properties of γ-Fe/Fe_3_C, including interfacial adhesion work, interfacial energy, and charge distribution, were also investigated systematically. Finally, we discussed the impact mechanism of Si on cementite nucleation, which provides theoretical support for the control of cementite nucleation during the solidification of hypereutectoid steel.

## 2. Experiment, Calculation Method, and Structure Model

### 2.1. Materials and Experimental Details

To verify the effect of Si on cementite nucleation, hypereutectoid steels with various Si concentrations were manufactured using a vacuum induction furnace. Ingots with various Si concentrations were obtained at the same cooling period and detected with a direct-reading spectrometer (ARL 3460 Thermo Fisher Scientific, Suzhou, China). The results are listed in Table 1.

In order to determine the phase of hypereutectoid steel with various Si concentrations, the phase precipitation of samples 0 #, 1 #, and 2 # was determined by JMatPro 9.0 thermodynamic software, as shown in Table 1. Furthermore, XRD (D8 ADVANCE) was performed on the three as-cast hypereutectoid steels. The scanning angle range was 10°–120°. Then, to investigate the influence of Si on the cementite nucleation, the cementite morphology of as-cast hypereutectoid steel with various Si concentrations was observed using SEM (Sigma 500 Zeiss, Erkrath, Germany). Twenty pictures of samples with various Si concentrations were randomly taken; the cementite lamellae numbers within the same area on multiple pictures were calculated by Image-Pro 6.0 software, and their average values were calculated. To observe the cementite lamellae more clearly, the samples with various Si concentrations were subjected to electrolytic corrosion using a mixed solution of acetic acid, perchloric acid, and distilled water (9:3:2). Using the sample as the anode, a direct current was continuously applied for 20 s with a constant voltage of 8 V.

In order to determine the orientation relationship between cementite and austenite, a quenching experiment was designed to retain the initial state of cementite precipitation. The experimental scheme was as follows. For 1 #, hypereutectoid steel was selected as the representative in the experiments. The sample was heated to 1000 °C under a vacuum with vacuum rapid quenching equipment and left for 2 h to make the sample completely austenitize. The furnace was cooled to 870 °C and held for 1 min. The sample was quickly cooled to room temperature with a quenching system. Finally, the quenched specimen was mechanically polished, and 0.05 μm aluminum oxide polishing solution was used for vibration polishing for 3 h. Then, the orientation relationship between Fe_3_C and γ-Fe was measured using EBSD (JSM6480, JEOL, Tokyo, Japan). The parameters of EBSD were set as an acceleration voltage of 20 kV, a working distance of 15 mm, and an inclination angle of 70°.

### 2.2. Calculation Parameters

The Vienna Ab initio Simulation Package was used for the first-principles calculations according to density functional theory [20,21]. The present work used two methods, the generalized gradient approximation (GGA) of the Perdew–Burke–Ernzerhof functional (PBE) [22] and the local density approximation (LDA) of Ceperley–Alder–Perdew–Zunger (CAPZ) [23], to calculate the bulk properties of γ-Fe and Fe_3_C, with the aim of increasing the accuracy of the calculation. In all the calculations of the surface properties and bulk properties of γ-Fe and Fe_3_C and the interfacial properties of γ-Fe/Fe_3_C, the cutoff energy was 400 eV. The K-points for the γ-Fe and Fe_3_C bulk γ-Fe (010)/Fe_3_C (010) interface models were set as 8 × 8 × 8, 6 × 5 × 4 and 4 × 6 × 1, respectively. A vacuum layer of 15 Å was added to the surface and interface models. When the difference was less than 1.0 × 10^−5^ eV/atom between the last two cycle energies of the self-consistent calculation, the system reached convergence. The maximum stress was 0.05 GPa, and the maximum ionic Hellmann–Feynman force was 0.03 eV/Å.

## 3. Results and Discussion

### 3.1. Phase Structure Analysis

**Figure 1:**
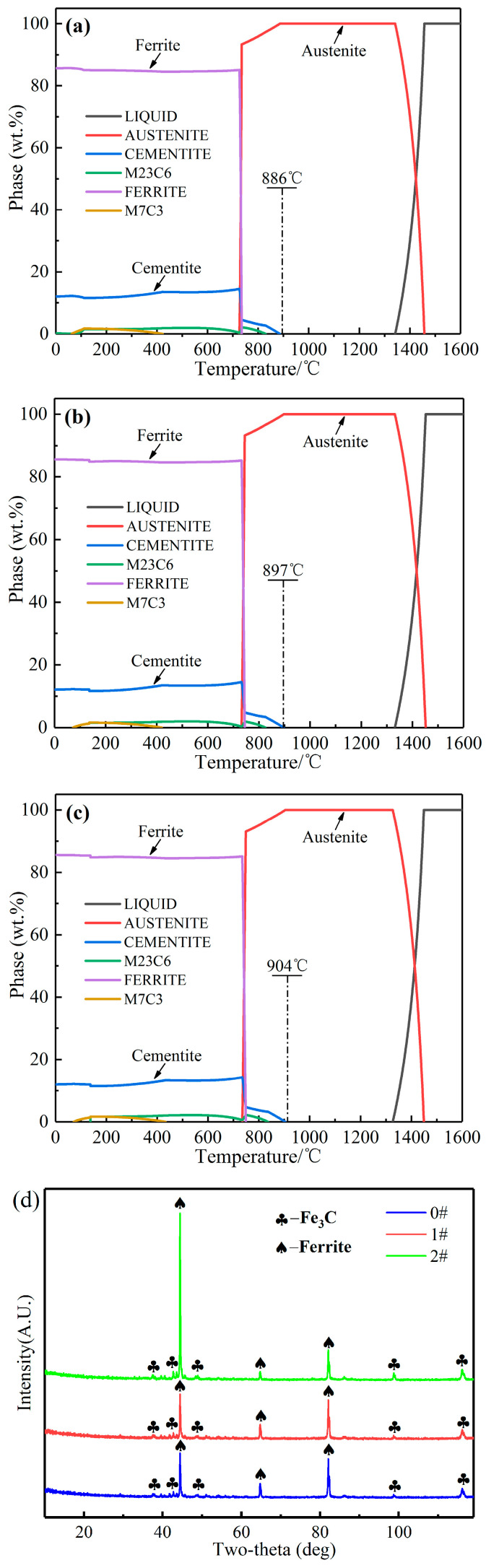
(**a**–**c**) Phase precipitation of samples 0 #, 1 # and 2 #; (**d**) XRD patterns of samples 0 #, 1 # and 2 #.

Figure 1a–c displays the thermodynamic results of three samples, 0 #–2 #. The results indicate that the change in Si concentrations has an insignificant effect on the phases of the three samples. The final solidification phase of the samples consists primarily of ferrite and cementite, with a small amount of M_7_C_3_ carbides. However, there is a variation in the precipitation temperature of cementite among three samples, with the temperatures for samples 0 #, 1 #, and 2 # being 886 °C, 897 °C, and 904 °C, respectively. This phenomenon indicates that an increase in Si concentrations induces a tendency for cementite precipitation at higher temperatures. Furthermore, based on the XRD detection results (Figure 1d), the phases of samples 0 #, 1 #, and 2 # are all α-Fe (ferrite) and Fe_3_C (cementite). The discrepancy between the XRD and the thermodynamic results may be attributed to the low concentrations of M_7_C_3_ carbide, which may not have detected the diffraction peak of M_7_C_3_ carbide by XRD.

### 3.2. Microstructure Analysis

**Figure 2:**
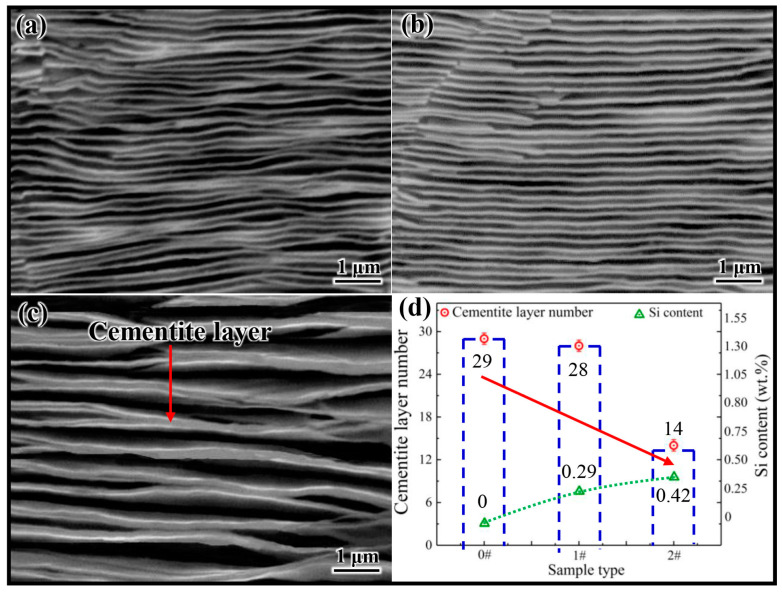
(**a**–**c**) Cementite microstructures of 0 #, 1 # and 2 #, respectively. (**d**) The relationship between the cementite lamellae number and the variation in Si content.

Figure 2a–c shows the microstructure of samples 0 #, 1 #, and 2 # after electrolytic corrosion, respectively. The gray lamellar structure in the figure represents the cementite. Comparing Figure 2a,c, it can be preliminarily determined that the number of cementite lamellae in sample 2 # is significantly lower than those in samples 0 # and 1 #. In order to obtain more accurate data, the number of cementite lamellae in 20 pictures of each sample was counted and averaged, and the statistical results are shown in Figure 2d. The results indicate that the Si concentration increased from 0 to 0.42 wt.%, resulting in a decrease in the average number of cementite lamellae per unit area from 29 to 14. This phenomenon suggests that the increase in Si concentration reduces the number of cementite nuclei per unit area in hypereutectoid steel, which also implies that Si has an inhibitory effect on cementite nucleation in hypereutectoid steel. However, it is difficult to reveal the essence of Si inhibiting cementite nucleation by experimental means. Therefore, in this study, based on the effect of Si on the austenite–cementite interface, the first principles calculations were applied to analyze the essence for Si inhibiting cementite nucleation from an atomic perspective.

### 3.3. First-Principle Calculations Analysis

#### 3.3.1. Bulk Properties of γ-Fe and Fe_3_C

Based on the thermodynamic results (Figure 2), cementite is the leader phase in hypereutectoid steel used in this experiment. Cementite nucleation preferentially occurs on austenite (γ-Fe). Therefore, the building of the austenite–cementite interfacial model is the key to exploring the effect of Si on the cementite nucleation. In addition, the XRD analysis confirms that the type of cementite present in hypereutectoid steel used in this experiment is Fe_3_C. Consequently, the interfacial model for austenite–cementite can be represented as γ-Fe/Fe_3_C.

The bulks that make up the γ-Fe/Fe_3_C interfacial model are γ-Fe and Fe_3_C, respectively. Table 2 shows the lattice parameters and volume (*V*) of the γ-Fe and Fe_3_C bulks. Comparing the calculation results of functions GGA (PBE) and LDA (CA-PZ) and the experimental values, it can be seen that the absolute errors between the calculated values (lattice parameters (a, b, and c), and *V*) of the Fe_3_C bulk obtained based using the GGA (PBE) function and the experimental values are 3.8%, 2.0%, 3.5%, and 9.2%, respectively. The absolute errors between the calculated values (lattice parameters (a, b, and c), and *V*) of the Fe_3_C bulk obtained using the LDA (CA-PZ) function and the experimental values are 5.5%, 4.2%, 5.4%, and 14.5%, respectively. Obviously, the calculation results of GGA (PBE) are closer to the experimental values than those of LDA (CA-PZ). Moreover, similar results mentioned above also exist in the calculation of Fe bulk. As a result, the GGA (PBE) function has a higher accuracy for the related calculations of γ-Fe and Fe_3_C and is adopted in subsequent calculations.

#### 3.3.2. Orientation Relationship between γ-Fe and Fe_3_C

**Figure 3:**
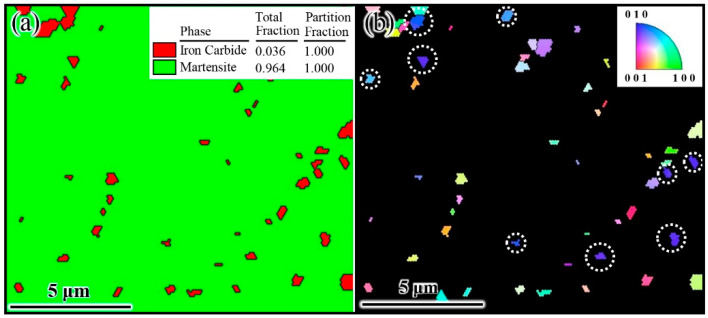
EBSD results of typical hypereutectoid steel. (**a**) Phase and (**b**) inverse pole figure (IPF).

Determining the orientation relationship between γ-Fe and Fe_3_C is the key to model building the γ-Fe/Fe_3_C. Thus, the initial state of cementite precipitation was obtained through quenching experiments, and the orientation relationship between γ-Fe and Fe_3_C was determined by EBSD, as shown in Figure 3. The red irregular block represents Fe_3_C, and the green part denotes martensite in Figure 3a. The reason for this phenomenon was the direct quenching from austenite and Fe_3_C, and the final structure was made of martensite and Fe_3_C. Because martensite and Fe_3_C are obtained through rapid cooling, the orientation relationship between martensite and Fe_3_C in EBSD can represent the orientation relationship between austenite (γ-Fe) and Fe_3_C in steel. The IPF shows that Fe_3_C was more likely to grow along the [010] orientation, as shown in Figure 3b (The position marked by the white circle in Figure 3b is [010] orientation). Furthermore, since Fe_3_C cooled rapidly to room temperature at the beginning of precipitation, its growth time was extremely short. The Fe_3_C orientation detected in Figure 3b can be identified as the orientation of Fe_3_C growth in austenite (γ-Fe). Thus, the phenomenon was determined as being due to Fe_3_C that grows in the [010] direction on the (010) crystal plane of austenite. This result was similar to those of Howe et al. and Zhang et al. [25,26]. Consequently, the model of the austenite–cementite composite structure was preliminarily determined to be γ-Fe (010)/Fe_3_C (010).

In addition, the lattice mismatch between the two surface models of γ-Fe (010) and Fe_3_C (010) was calculated. The lattice parameters of the Fe_3_C (010) surface model are a = 4.353 Å and b = 6.593 Å. In order to match the interface of Fe_3_C (010), γ-Fe (010) was set with a 1 × 2 supercell, and the lattice parameters of γ-Fe (010) after the supercell are a = 3.435 Å and b = 6.870 Å. According to lattice mismatch calculations, the lattice mismatch between the Fe_3_C (010) (1 × 1) and γ-Fe (010) (1 × 2) surfaces is 8.6%. Bramfitt indicates that if the lattice mismatch is less than 12%, and the nucleation of grains is effective [27]. Therefore, γ-Fe (010) is effective as the nucleation core of Fe_3_C (010), and the final austenite–cementite interface model was determined as γ-Fe (010)/Fe_3_C (010) in this study.

#### 3.3.3. Surface Properties of γ-Fe (010) and Fe_3_C (010)

The convergence testing of the slicing layers of the γ-Fe (010) slab and Fe_3_C (010) slab is necessary before establishing the interface model to ensure that the interior of the γ-Fe (010) slab and the Fe_3_C (010) slab can achieve bulk properties.

The surface energy *σ*_γ-Fe (010)_ of the γ-Fe (010) slab is expressed as:

(1) $$\sigma_{\text{γ-Fe}\;(010)}=\frac{1}{2A_{\mathrm{Fe}\;(010)}}[E_{\text{γ-Fe}\;(010)}^{\mathrm{slab}}-N_{\mathrm{Fe}}^{\text{γ-Fe}\;(010)}\mu_{\mathrm{Fe}}^{\mathrm{bulk}}]$$

(2) $$\mu_{\mathrm{Fe}}^{\mathrm{bulk}}=E_{\mathrm{Fe}}^{\mathrm{bulk}}/n_{\mathrm{Fe}}$$

where *A*_Fe (010)_ is the cross-sectional area of the γ-Fe (010) slab; $E_{\text{γ-Fe}\;(010)}^{\mathrm{slab}}$ is the total energy of γ-Fe (010) slab; $N_{\mathrm{Fe}}^{\text{γ-Fe}\;(010)}$ is the number of Fe atoms in the γ-Fe (010) slab; $\mu_{\mathrm{Fe}}^{\mathrm{bulk}}$ is the chemical potential of the Fe atom in Fe bulk; $E_{\mathrm{Fe}}^{\mathrm{bulk}}$ is the total energy of Fe bulk; and *n*_Fe_ is the number of Fe atoms in the Fe bulk.

Integrating Equations (1) and (2), we derive

(3) $$\sigma_{\text{γ-Fe}\;(010)}=\frac{1}{2A_{\mathrm{Fe}(010)}}[E_{\text{γ-Fe}\;(010)}^{\mathrm{slab}}-N_{\mathrm{Fe}}^{\text{γ-Fe}\;(010)}\;E_{\mathrm{Fe}}^{\mathrm{bulk}}/n_{\mathrm{Fe}}]$$

**Figure 4:**
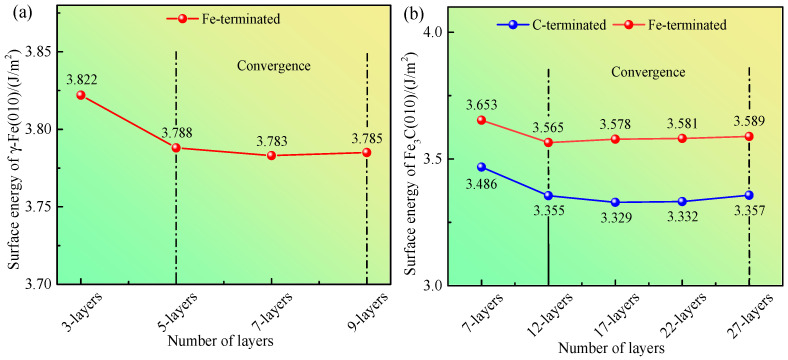
(**a**) Calculated surface energy of γ-Fe (010). (**b**) Calculated surface energy of Fe_3_C (010).

The values of *σ*_γ-Fe (010)_ calculated from Equation (3) are shown in Figure 4a. When the number of layers reaches five, the σ_γ-Fe(010)_ of the γ-Fe (010) slab tends to be constant. These results indicate that when the atomic layer thickness is five slicing layers, the γ-Fe (010) slab reaches the convergence state.

The surface energy $\sigma_{{\mathrm{Fe}}_{3}C\;(010)}$ of the Fe_3_C (010) slab is expressed as:

(4) $$\sigma_{{\mathrm{Fe}}_{3}C\;(010)}=\frac{1}{2A_{{\mathrm{Fe}}_{3}C\;(010)}}[E_{{\mathrm{Fe}}_{3}C\;(010)}^{\mathrm{slab}}-N_{\mathrm{Fe}}^{{\mathrm{Fe}}_{3}C\;(010)}\;\mu_{\mathrm{Fe}}^{\mathrm{slab}}-N_{C}^{{\mathrm{Fe}}_{3}C\;(010)}\;\mu_{C}^{\mathrm{slab}}]$$

where $A_{{\mathrm{Fe}}_{3}C\;(010)}$ is the cross-sectional area of the Fe_3_C (010) slab; $E_{{\mathrm{Fe}}_{3}C}^{\mathrm{slab}}$ is the total energy of Fe_3_C (010) slab; $N_{\mathrm{Fe}}^{{\mathrm{Fe}}_{3}C\;(010)}$ is the number of Fe atoms in the Fe_3_C (010) slab; $N_{C}^{{\mathrm{Fe}}_{3}C\;(010)}$ is the number of C atoms in the Fe_3_C (010) slab; $\mu_{\mathrm{Fe}}^{\mathrm{slab}}$ is the chemical potential of the Fe atom in Fe_3_C; and $\mu_{C}^{\mathrm{slab}}$ is the chemical potential of the C atom in Fe_3_C.

The total energy $\mu_{{\mathrm{Fe}}_{3}C}^{\mathrm{bulk}}$ of bulk Fe_3_C is

(5) $$\mu_{{\mathrm{Fe}}_{3}C}^{\mathrm{bulk}}=3\mu_{\mathrm{Fe}}^{\mathrm{slab}}+\mu_{C}^{\mathrm{slab}}$$

There are two types of surfaces in the Fe_3_C (010) slab—C-terminated and Fe-terminated. Thus, the Fe_3_C (010) slab has two types of surface energy: $\sigma_{{\mathrm{Fe}}_{3}C\;(010)\;(\text{Fe-terminated})}$ and $\sigma_{{\mathrm{Fe}}_{3}C\;(010)\;(\text{C-terminated})}$. Integrating Equations (4) and (5), we derive

(6) $$\sigma_{{\mathrm{Fe}}_{3}C\;(010)\;(\text{Fe-terminated})}=\frac{1}{2A_{{\mathrm{Fe}}_{3}C\;(010)}}[E_{{\mathrm{Fe}}_{3}C\;(010)}^{\mathrm{slab}}-N_{C}^{{\mathrm{Fe}}_{3}C(010)}\;\mu_{{\mathrm{Fe}}_{3}C}^{\mathrm{bulk}}+(3\;N_{C}^{{\mathrm{Fe}}_{3}C\;(010)}-N_{\mathrm{Fe}}^{{\mathrm{Fe}}_{3}C\;(010)})\;\mu_{\mathrm{Fe}}^{\mathrm{slab}}]$$

(7) $$\sigma_{{\mathrm{Fe}}_{3}C\;(010)\;(\text{C-terminated})}=\frac{1}{2A_{{\mathrm{Fe}}_{3}C\;(010)}}[E_{{\mathrm{Fe}}_{3}C\;(010)}^{\mathrm{slab}}-N_{C}^{{\mathrm{Fe}}_{3}C(010)}\;\mu_{{\mathrm{Fe}}_{3}C}^{\mathrm{bulk}}/3+(N_{\mathrm{Fe}}^{{\mathrm{Fe}}_{3}C\;(010)}/3-N_{C}^{{\mathrm{Fe}}_{3}C\;(010)})\;\mu_{C}^{\mathrm{slab}}]$$

To evaluate the convergence, $\mu_{\mathrm{Fe}}^{\mathrm{slab}}$ ≈ $\mu_{\mathrm{Fe}}^{\mathrm{bulk}}$ = −865.194 eV is taken, $\mu_{C}^{\mathrm{slab}}$ ≈ $\mu_{C}^{\mathrm{bulk}}$ = −155.079 eV is taken, where $\mu_{C}^{\mathrm{bulk}}$ is the chemical potential of the C atom in the C bulk. The values of $\sigma_{{\mathrm{Fe}}_{3}C\;(010)\;(\text{Fe-terminated})}$ and $\sigma_{{\mathrm{Fe}}_{3}C\;(010)\;(\text{C-terminated})}$ calculated using Equations (6) and (7) are shown in Figure 4b. When the number of layers reaches 12, the $\sigma_{{\mathrm{Fe}}_{3}C\;(010)\;(\text{Fe-terminated})}$ and $\sigma_{{\mathrm{Fe}}_{3}C\;(010)\;(\text{C-terminated})}$ tend to be constant. These results demonstrate that when the atomic layer thickness is 12 slicing layers, the Fe_3_C (010) slab reaches the convergence state.

The chemical potential was approximated in the above calculations to determine the convergence of the surface model. Actually, $\mu_{\mathrm{Fe}}^{\mathrm{slab}}$ and $\mu_{C}^{\mathrm{slab}}$ are variables. In order to determine the terminal type of the Fe_3_C (010) slab with a thickness of 12 slicing layers, according to the literature [28], the heat of formation Δ$H_{f}^{0}$(Fe_3_C) for the bulk Fe_3_C was introduced, and the formula is as follows:

(8) $$\Delta H_{f}^{0}({\mathrm{Fe}}_{3}C)=\mu_{{\mathrm{Fe}}_{3}C}^{\mathrm{bulk}}-3\mu_{\mathrm{Fe}}^{\mathrm{bulk}}-\mu_{C}^{\mathrm{bulk}}$$

Thus, Δ$H_{f}^{0}$(Fe_3_C) = −2.73 eV. Integrating Equations (5) and (8), we derive:

(9) $$\mu_{C}^{\mathrm{slab}}-\mu_{C}^{\mathrm{bulk}}=\Delta H_{f}^{0}({\mathrm{Fe}}_{3}C)-3(\mu_{\mathrm{Fe}}^{\mathrm{slab}}-\mu_{\mathrm{Fe}}^{\mathrm{bulk}})$$

Given that $\mu_{C}^{\mathrm{slab}}$ − $\mu_{C}^{\mathrm{bulk}}$ ≤ 0 and $\mu_{\mathrm{Fe}}^{\mathrm{slab}}$ − $\mu_{\mathrm{Fe}}^{\mathrm{bulk}}$ ≤ 0, we obtain the following relationship:

(10) $$\Delta H_{f}^{0}({\mathrm{Fe}}_{3}C)\leq \mu_{C}^{\mathrm{slab}}-\mu_{C}^{\mathrm{bulk}}\leq 0$$

**Figure 5:**
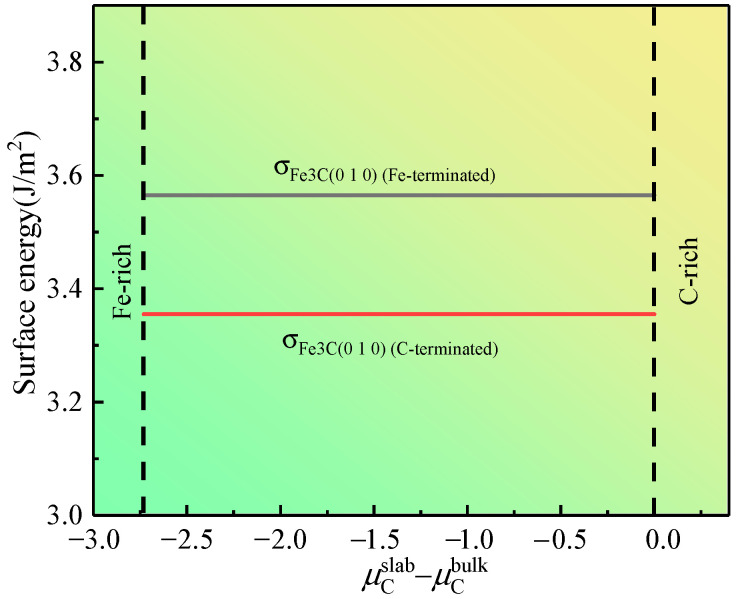
Surface energy of the 12-layered Fe_3_C (010) slab as a function of $\mu_{C}^{\mathrm{slab}}$ − $\mu_{C}^{\mathrm{bulk}}$.

Integrating Equations (6), (7), and (10), we determine the relationship between $\sigma_{{\mathrm{Fe}}_{3}C\;(010)}$ for the 12-layer Fe_3_C (010) slab and $\mu_{C}^{\mathrm{slab}}$ − $\mu_{C}^{\mathrm{bulk}}$, as shown in Figure 5. The figure indicates that $\sigma_{{\mathrm{Fe}}_{3}C\;(010)\;(\text{C-terminated})}$ is smaller than $\sigma_{{\mathrm{Fe}}_{3}C\;(010)\;(\text{Fe-terminated})}$ in the whole range of $\mu_{C}^{\mathrm{slab}}$ − $\mu_{C}^{\mathrm{bulk}}$. This finding implies that the stability of the Fe_3_C (010) slab with C-terminated is better from the perspective of thermodynamics. Thus, the cementite–austenite interface model is established using the C-terminated Fe_3_C (010) slab and γ-Fe (010) slab. In addition, Matthew et al. [29] emphasized that modeling in the form of FeC-Fe (C is the termination in cementite; Fe is the termination in γ-Fe) exhibits lower interfacial energy than that in the form of Fe-FeC (Fe is the termination in cementite; Fe is the termination in γ-Fe).

#### 3.3.4. γ-Fe (010)/Fe_3_C (010) Interface Models

**Figure 6:**
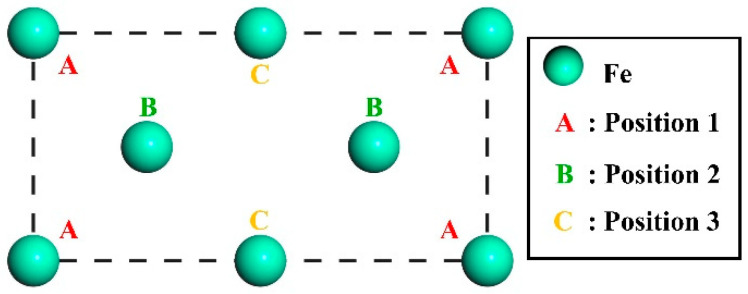
Atomic layout of the γ-Fe (010) crystal face.

**Figure 7:**
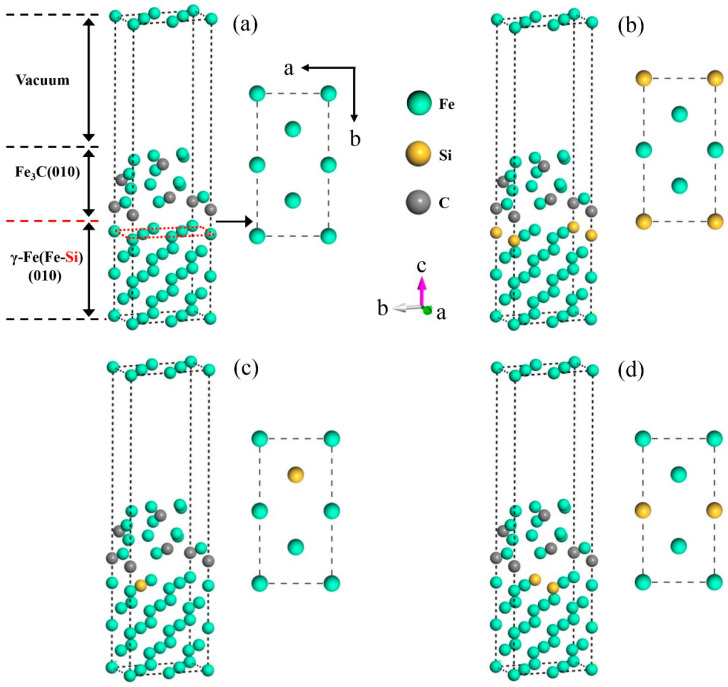
γ-Fe (010)/Fe_3_C (010) models with different Si atom doping sites. (**a**) Clean, (**b**) Structure A, (**c**) Structure B, and (**d**) Structure C.

To reveal the influence of Si on the interface properties of γ-Fe (010)/Fe_3_C (010), Si atoms must be doped at the interface of γ-Fe (010)/Fe_3_C (010). However, Si is a non-carbide-forming element. When the cementite precipitates, Si atoms migrate to austenite around cementite and exist in the shape of a displacement solution [30]. Hence, in the model, the doping mode of Si atoms aims to replace the Fe atoms on the austenite side of the interface. According to Section 3.3.2, austenite and cementite were connected by the (010) crystal surface. The (010) slab was cut on the austenite primitive cell. The atomic structure on the surface of the slab is shown in Figure 6. The γ-Fe (010) slab has a symmetrical structure and three doping sites, A, B, and C (corresponding to positions 1, 2, and 3, respectively), on the surface. Therefore, the Si atoms replace Fe atoms at the three positions mentioned above, and the three models were established, named Structures A–C, as shown in Figure 7. In addition, the model without Si atoms was named Clean.

#### 3.3.5. γ-Fe (010)/Fe_3_C (010) Interface Properties

The work of adhesion and interfacial energy are two significant parameters for judging the bonding strength and stability of interfaces, and they make a difference in evaluating the properties of each phase interface. The work of adhesion represents the adhesion strength of the phase interface and denotes the reversible work required to form two free surfaces per unit area. The greater the work of adhesion is, the better the stability is between the two phases. The expression of the work of adhesion is [31,32,33]

(11) $$W_{\mathrm{ad}}=\;\frac{1}{A}\;\left(E_{\text{γ-Fe}\;(010)}\;+\;E_{{\mathrm{Fe}}_{3}C\;(010)}\;+\;E_{\text{γ-Fe}\;(010)/{Fe}_{3}C\;(010)}\right)$$

where $E_{\text{γ-Fe}\;(010)/{\mathrm{Fe}}_{3}C\;(010)}$ is the total energy of the γ-Fe (010)/Fe_3_C (010) model; *E*_γ-Fe(010)_ and $E_{{\mathrm{Fe}}_{3}C\;(010)}$ are the total energies of the γ-Fe (010) slab and Fe_3_C (010) slab, respectively; and *A* is the interface area of the γ-Fe (010)/Fe_3_C (010) model.

The interfacial energy represents the superfluity energy per unit area at the phase interface and plays an important role in the characterization of interface stability. A lower interfacial energy can represent a higher stability between the two phases. The expression for interfacial energy is [34,35]

(12) $$\gamma_{\mathrm{int}}=\sigma_{\text{γ-Fe}\;(010)}+\sigma_{{\mathrm{Fe}}_{3}C\;(010)}-W_{\mathrm{ad}}$$

where *σ*_γ-Fe(010)_ denotes the surface energy of the γ-Fe (010) slab; and $\sigma_{{\mathrm{Fe}}_{3}C\;(010)}$ is the surface energy of the Fe_3_C (010) slab.

#### 3.3.6. Work of Adhesion and Interfacial Energy

**Figure 8:**
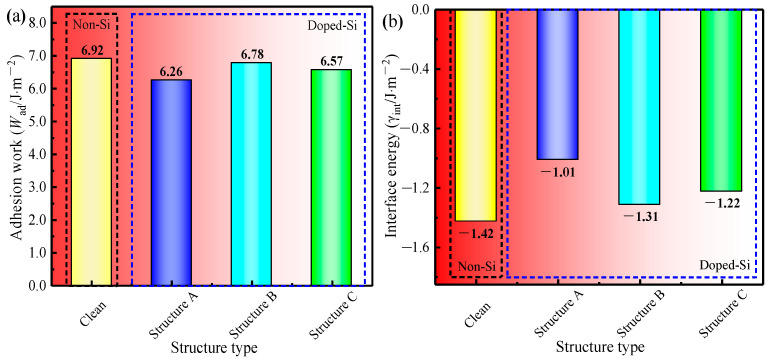
(**a**) W_ad_ of four models; (**b**) γ_int_ of four models.

According to Equation (11), the *W*_ad_ of the four models in Figure 7 was obtained, and the computed results are shown in Figure 8a. When the Si atom was doped, the *W*_ad_ of Structure B was the largest (6.78 J·m^−2^), which implies that the bonding interfacial bonding strength of γ-Fe (010)/Fe_3_C (010) was stronger when the Si atom was doped at Position 2. One possible reason is that compared with the distance between other doping positions on the γ-Fe (010) slab and the nearest atom on the Fe_3_C side, the distance between the atom at Position 2 and the nearest atom on the Fe_3_C side is greater (Table 3). Therefore, the effect of doping Si atoms at Position 2 on the γ-Fe (010)/Fe_3_C (010) interface was relatively minimal, which made the decrease in *W*_ad_ at the γ-Fe (010)/Fe_3_C (010) interface slight compared with those of the undoped Si atoms. A similar phenomenon has also been found in previous studies on the adhesion work of Al(111)/Al_3_BC(0001) interfaces [36]. In addition, according to Equation (12), the *γ*_int_ of the interface of the four models in Figure 7 was obtained, as shown in Figure 8b. Among Structures A-D with Si doping, the interfacial energy of Structure B was the lowest (−1.31 J·m^−2^), which meant that the Si atom was replaced with an Fe atom at Position 2 of the γ-Fe (010) slab, and the stability of the γ-Fe (010)/Fe_3_C (010) interface was better. Considering the *W*_ad_ and *γ*_int_ of the γ-Fe (010)/Fe_3_C (010) interface with Si doping, the occupation of Si on the γ-Fe (010) slab was more inclined to Position 2. Therefore, when further considering the influence of Si on the stability of the γ-Fe (010)/Fe_3_C (010) model, we should compare it with Clean and Structure B.

Comparing the *W*_ad_ of Clean and Structure B, that of Structure B (6.78 J·m^−2^) is lower than that of Clean (6.92 J·m^−2^). Regarding the *γ*_int_ of the two models, Structure B (−1.31 J·m^−2^) is greater than Clean (−1.42 J·m^−2^). The results indicate that the doping of Si at the γ-Fe (010)/Fe_3_C (010) interface reduces the interfacial stability of the γ-Fe (010)/Fe_3_C (010) model. In addition, the results are consistent with the thermodynamic research results indicating that Si reduces the driving force of cementite nucleation [9]. The inhibition effect of Si on cementite nucleation must be further analyzed at the atomic level.

#### 3.3.7. Charge Distribution and Density of States

**Figure 9:**
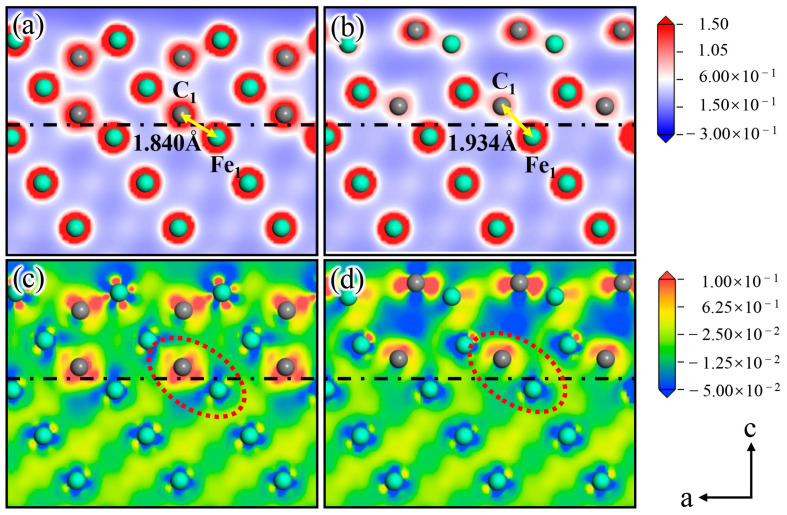
(**a**,**c**) show the charge density and the charge density difference of Clean, and (**b**,**d**) show the charge density and the charge density difference of Structure B.

To reveal the influence of Si on the interface stability of the γ-Fe (010)/Fe_3_C (010) composite structure at a deeper level, we analyzed the essence from the viewpoints of charge distribution and electron transfer. In addition, to clearly show the corresponding relationship between the Fe and C atoms above and below the interface, the a–c two-dimensional was chosen as the exploration target of the interfacial charge distribution of Clean and Structure B in Figure 9a–d.

Figure 9a shows the charge density of Clean, in which a strong bond is formed between C_1_ and Fe_2_ at the interface, and the distance between the two atoms is 1.840 Å. Based on the analysis of the charge density difference of Clean (Figure 9c), the charge distribution shows obvious localized characteristics at the interface (As indicated by the red circle). The charge of the Fe_2_ atom on the austenite side is lost, and the C_1_ atom on the cementite side acquires a substantial amount of charge. Figure 9b shows the charge density of the Si atom doped at Position 2. C_1_ and Fe_2_ are also bonded, but the distance between the two atoms is 1.934 Å, which is larger than that of two atoms at the same site before Si atom doping. The analysis of the charge density difference of Structure B (Figure 9d) suggests that the charge around C_1_ in Figure 9d exhibits apparent asymmetry compared with that of C_1_ in Figure 9c. Moreover, the charge density between C_1_ and Fe_2_ decreases significantly, reducing the binding ability between the two atoms [37,38]. This occurrence also explains the increase in the C_1_-Fe_2_ bond length after Si atom doping.

In addition, the literature indicates that Si atoms have gravitation on Fe atoms and repulsion on the C atoms [39]. At the austenite–cementite interface, C is terminated on the cementite side, and Fe is terminated on the austenite side. Therefore, Si atoms at the interface attract Fe atoms on the austenite side, whereas they repel C atoms on the cementite side. The charge distribution was disrupted at the interface due to the abovementioned effect of the Si atom, as presented in Figure 9c,d. In addition, the aforementioned effect in which the Si atom changes the interfacial charge transfer and reduces the atomic interactive force on both sides of the interface explains why doping the Si atom at Position 2 on the γ-Fe (010) slab reduces the γ-Fe (010)/Fe_3_C (010) interfacial stability.

**Figure 10:**
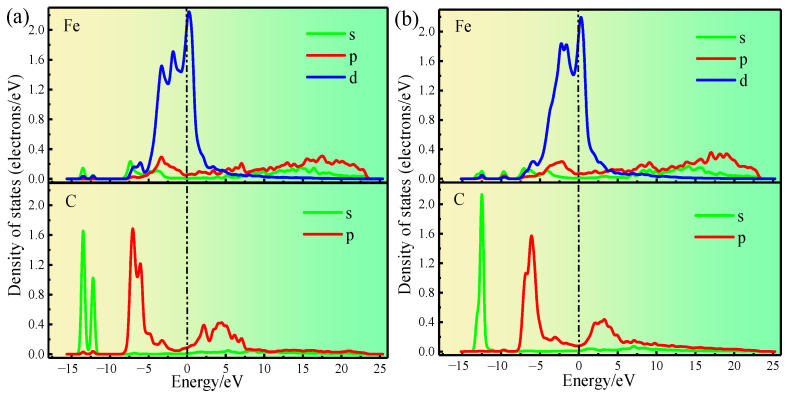
(**a**) shows the partial density of states of Clean. (**b**) shows the partial density of states of Structure B.

The partial density of states of C_1_ and Fe_1_ of Clean and Structure B were analyzed (Figure 10a,b) to understand the influence of Si on electron transfer between C_1_ and Fe_1_ more clearly. In Clean (Figure 10a), the Fe_1_-d and C_1_-p orbitals are hybridized at the electronic states −3.2 eV, −6.0 eV, and −7.0 eV; moreover, the Fe_1_-s and C_1_-s orbitals are hybridized at the electronic states −12.2 and −13.5 eV. In Structure B (Figure 10b), the Fe_1_-d and C_1_-p orbitals are hybridized at the electronic states −3.0 and −6.0 eV, and the Fe_1_-s and C_1_-s orbitals are hybridized at the electronic state −12.4 eV. Comparing the partial density of states of the C_1_ and Fe_1_ atoms before and after Si atom doping, the quantity of orbital hybridization of the Fe_1_-d and C_1_-p is decreased at the electron state from 0 to −8 eV as a result of Si atom doping. Meanwhile, at the electron state from −10 eV to −15 eV, the orbital hybridization of Fe_1_-s and C_1_-s is reduced to one. The weakening of electronic orbital hybridization between two atoms indicates a decrease in their interaction forces [37]. Consequently, the fundamental reason why Si atoms increase the distance between Fe_1_ and C_1_ is that doping with Si atoms reduces the number of electrons transferred between Fe_1_ and C_1_, ultimately leading to a decrease in interatomic interactions. Moreover, this result strongly proves that Si atom doping reduces the interfacial stability of γ-Fe (010)/Fe_3_C (010) from the perspective of electron transfer.

### 3.4. Inhibition Mechanism of Si on Fe_3_C Nucleation on γ-Fe

**Figure 11:**
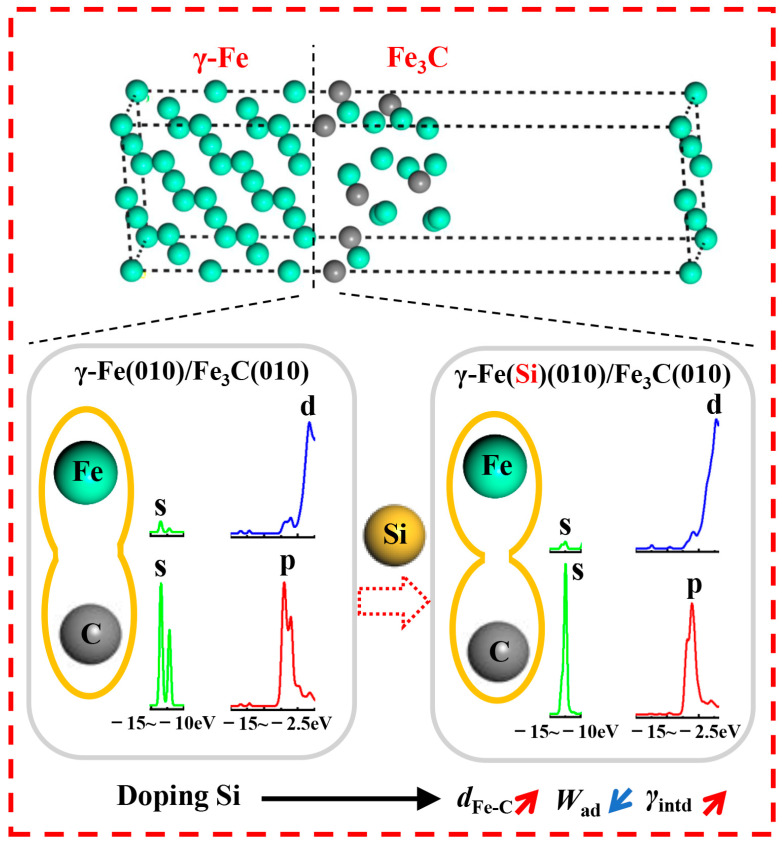
The impact mechanism of Si on Fe_3_C nucleation, where d_Fe-C_ is the distance between Fe and C atoms.

Based on the interfacial properties of the γ-Fe (010)/Fe_3_C (010) composite structure (the work of adhesion, interfacial energy, charge distribution, and density of states), the possible inhibition nucleation mechanism of Si on Fe_3_C on the γ-Fe matrix is shown in Figure 11. The existence of Si atoms weakens the electron transfer of atoms at the γ-Fe (010)/Fe_3_C (010) structure interface, breaking the balance of the charge distribution. Thus, the interaction force between the atoms on the γ-Fe (010) side and Fe_3_C (010) side is reduced, and the distance between the atoms is increased. The effects of Si directly lead to a decrease in the *W*_ad_ of the γ-Fe/Fe_3_C interface and an increase in γ_int_. Therefore, the interfacial stability of the γ-Fe/Fe_3_C interface decreased. According to the minimum energy theory, the direction of natural changes in the system is to reduce energy. Thus, when Si exists, Fe_3_C cannot easily nucleate in the existing γ-Fe to form the γ-Fe/Fe_3_C composite structure. Furthermore, the previous research indicated that the essence of cementite nucleation was the reconstructive transformation of the original positions for Fe, C, and substitutional elements, which may only be related to atomic migration [40,41]. Additionally, Babu at el. indicated that atom probe analysis failed to detect concentration spikes of substitutional elements at the matrix–cementite interface, and these results were also confirmed by Chang and Smith [42]. Consequently, the atomic migration of Fe and C was one of the leading factors of cementite nucleation, which convincingly demonstrates the following possible conclusion. The essence of the influence of Si on Fe_3_C nucleation is that Si attenuates the interaction force between nearby Fe and C atoms, thus reducing the migration and reconstruction of Fe and C atoms in the original configuration. Moreover, the aforementioned theory also explains why the presence of Si, as mentioned in the literature, weakens the driving force of cementite nucleation [9].

## 4. Conclusions

(1) An increase in Si concentration (0–0.42 wt.%) has an insignificant effect on the types of cementite in as-cast hypereutectoid steel. However, the average number of cementite nuclei per unit area decreased obviously.
(2) Even if the Si atom occupied the optimal site in the interface (position 2 in the γ-Fe (010) slab), the stability of γ-Fe (010)/Fe_3_C (010) was reduced, which is reflected in the decrease in the work of adhesion (from 6.92 J·m^−2^ to 6.78 J·m^−2^) and the increase in the interfacial energy (from −1.42 J·m^−2^ to −1.31 J·m^−2^).
(3) The interfacial charge distribution was disrupted when the Si atoms were doped at the interface of γ-Fe (010)/Fe_3_C (010). The partial density of states indicate that the Si atoms decreased the hybridization of Fe and C orbits on both sides of the interface at the electronic states from 0 to −8 eV and from −10 eV to −15 eV, respectively.
(4) In conclusion, at the γ-Fe (010)/Fe_3_C (010) interface, the doping of Si atoms weakened the electronic transfer between Fe and C atoms and decreased the interactive force between atoms on the γ-Fe side and Fe_3_C side, which led to less interfacial stability. This phenomenon clarified the fundamental reason for Si inhibiting the nucleation ability of cementite.

## Figures and Tables

**Table 1:** Composition of hypereutectoid steel with various Si concentrations (wt.%).

Type	C	Si	Mn	Ni	Cr	Mo	Al	Fe
0 #	1.047	0.088	0.320	0.155	1.529	0.293	0.0067	Balance
1 #	0.985	0.292	0.335	0.159	1.509	0.319	0.0012	Balance
2 #	1.049	0.425	0.319	0.162	1.575	0.333	0.0034	Balance

**Table 2:** Calculated bulk properties of γ-Fe and Fe_3_C.

Phases	Method	a (Å)	b (Å)	c (Å)	α = β = γ (°)	*V* (Å^3^)
Fe_3_C	GGA (PBE)	4.353	6.593	4.889	90	140.311
	LDA (CA-PZ)	4.268	6.449	4.801	90	132.144
	Experiment [24]	4.514	6.733	5.083	90	154.486
γ-Fe	GGA (PBE)	3.435	3.435	3.435	90	40.530
	LDA (CA-PZ)	3.361	3.361	3.361	90	37.966
	Experiment [24]	3.585	3.585	3.585	90	46.075

**Table 3:** The distance between the doping positions on the γ-Fe (010) slab and the nearest atom on the Fe_3_C side.

Doping Sites of Si Atom	Position 1	Position 2	Position 3
Distance	2.559 Å	3.083 Å	3.014 Å

## Data Availability

Data are contained within the article.
